# Supplementary material for: Retinotopic connectivity maps of human visual cortex with unconstrained eye movements
Source: Hum Brain Mapp. 2023 Aug 9;44(16):5221–37. doi: 10.1002/hbm.26446 (PMC10543111; doi:10.1002/hbm.26446)
Supplement: Supplementary file 1 — Figure S1. The panels show the mean intercept (A,C) and slope (B,D) coefficients from linear regression between eccentricities and inhibitory CF size (A,B) and suppression strength (C,D), respectively. Symbols and error bars denote the group mean parameter ±1 standard error. Violin plots show the bootstrapped distribution smoothed with a kernel function. For inhibitory CF size, data were log‐transformed before linear regression. Note that because V1 was used as template region, the peak facilitatory correlations in this region are self‐referential. Nevertheless, these data permit a meaningful estimate of the inhibitory subfields in this region. Figure S2. The effect of large eye movements on retinotopic maps in the Unstable Eye experiment of all subjects participated in the task (P4–P6). Polar angle (left) and eccentricity maps (right) were derived by pRF and CF analysis (rows), either with stable or random fixation patterns (columns). Each panel shows maps of given participant. The gray bars denote the participant identified and their refractive error in the left (L) and right (R) eyes. Note that V1 was the template region for CF analysis. CF estimates in this region are necessarily self‐referential and simply quantify how strongly the time series of a given vertex correlates with those of its neighbors. Including V1 however helps orient the reader and facilitates a direct comparison of the coherence of CF and pRF maps between the two experimental conditions. [file HBM-44-5221-s001.docx]

**Supplementary Information**


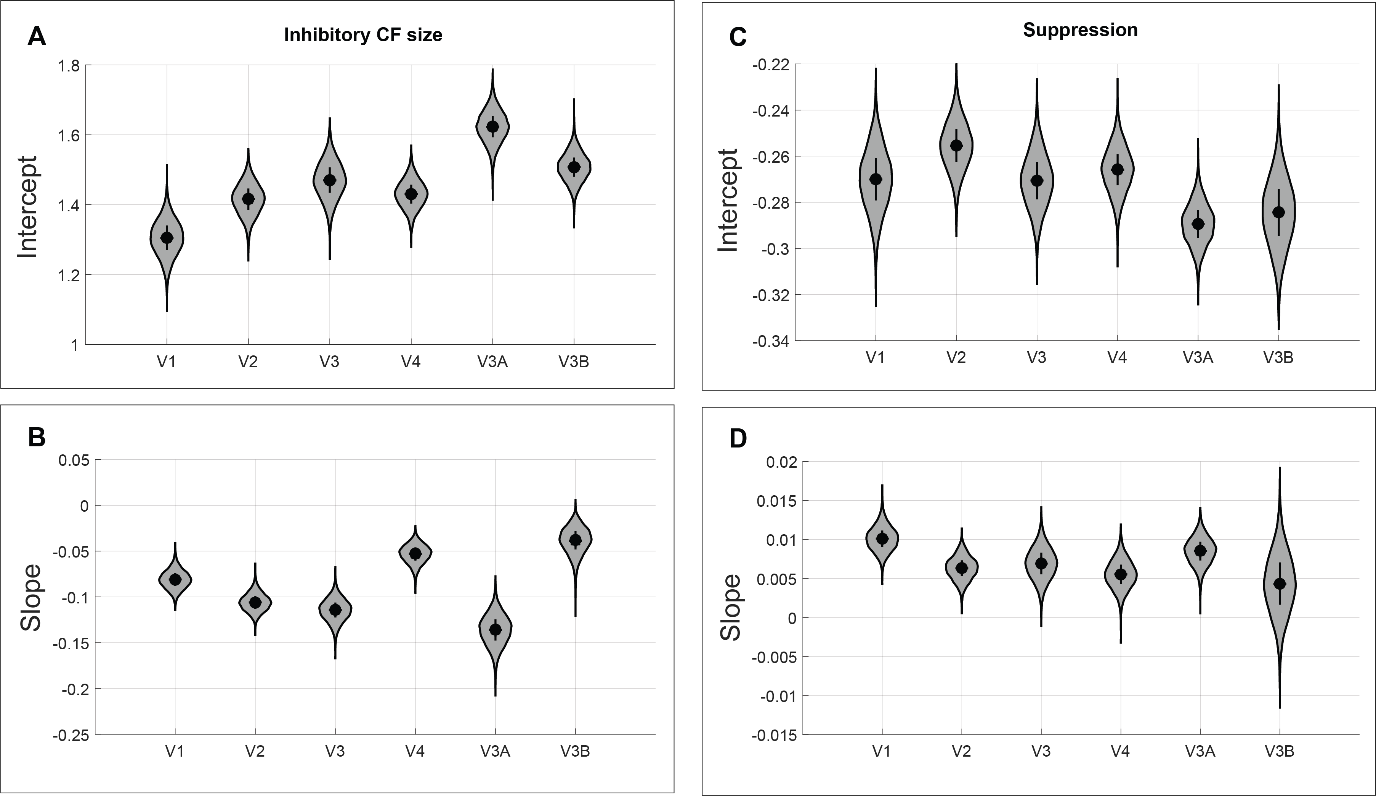


**Supplementary Figure S1.** The panels show the mean intercept (**A,C)** and slope **(B,D)** coefficients from linear regression between eccentricities and inhibitory CF size (**A,B**) and suppression strength (**C,D**), respectively. Symbols and error bars denote the group mean parameter ±1 standard error. Violin plots show the bootstrapped distribution smoothed with a kernel function. For inhibitory CF size, data were log-transformed before linear regression. Note that because V1 was used as template region, the peak facilitatory correlations in this region are self-referential. Nevertheless, these data permit a meaningful estimate of the inhibitory subfields in this region.


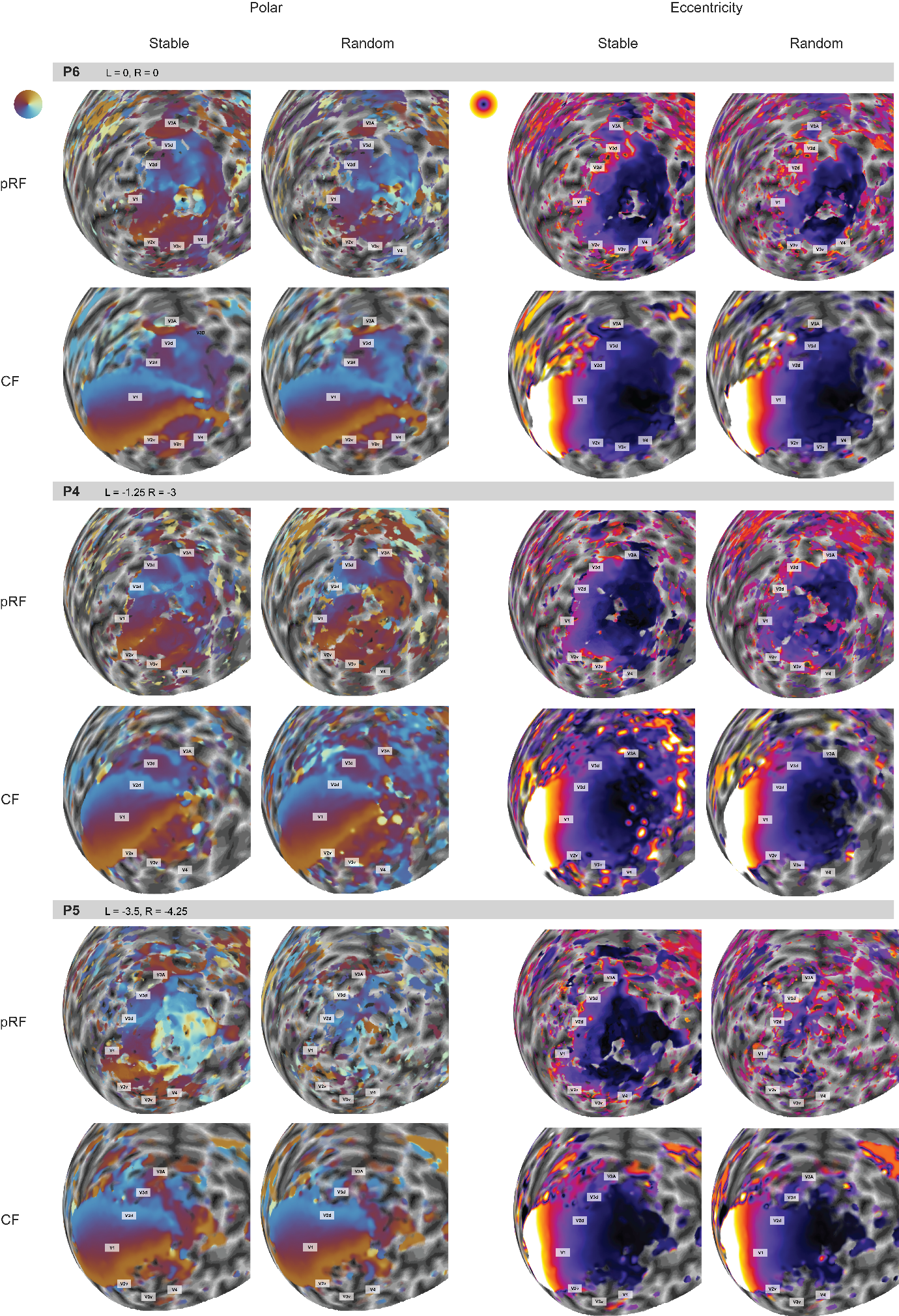


**Supplementary Figure S2.** The effect of large eye movements on retinotopic maps in the *Unstable Eye* experiment of all subjects participated in the task (P4-P6). Polar angle (left) and eccentricity maps (right) were derived by pRF and CF analysis (rows), either with stable or random fixation patterns (columns). Each panel shows maps of given participant. The gray bars denote the participant identified and their refractive error in the left (L) and right (R) eyes. Note that V1 was the template region for CF analysis. CF estimates in this region are necessarily self-referential and simply quantify how strongly the time series of a given vertex correlates with those of its neighbors. Including V1 however helps orient the reader and facilitates a direct comparison of the coherence of CF and pRF maps between the two experimental conditions.
